# Empathy, Altruism, and Group Identification

**DOI:** 10.3389/fpsyg.2021.749315

**Published:** 2021-12-14

**Authors:** Kengo Miyazono, Kiichi Inarimori

**Affiliations:** ^1^Graduate School of Humanities and Human Sciences, Hokkaido University, Sapporo, Japan; ^2^Center for Human Nature, Artificial Intelligence, and Neuroscience, Hokkaido University, Sapporo, Japan

**Keywords:** empathy, empathic emotion, altruism, group identification, self-other merging

## Abstract

This paper investigates the role of group identification in empathic emotion and its behavioral consequences. Our central idea is that group identification is the key to understanding the process in which empathic emotion causes helping behavior. Empathic emotion causes helping behavior because it involves group identification, which motivates helping behavior toward other members. This paper focuses on a hypothesis, which we call “self-other merging hypothesis (SMH),” according to which empathy-induced helping behavior is due to the “merging” between the helping agent and the helped agent. We argue that SMH should be interpreted in terms of group identification. The group identification interpretation of SMH is both behaviorally adequate (i.e., successfully predicts and explains the helping behavior in the experimental settings) and psychologically plausible (i.e., does not posit psychologically unrealistic beliefs, desires, etc.). Empathy-induced helping behavior, according to the group identification interpretation of the SMH, does not fit comfortably into the traditional egoism/altruism dichotomy. We thus propose a new taxonomy according to which empathy-induced helping behavior is both altruistic at the individual level and egoistic at the group level.

## Introduction

This paper investigates the role of group identification (which is, roughly, the process by which one acquires a form of self-conception as a group member) in empathic emotion and its behavioral consequences. Our central idea is that group identification is the key to understanding the process in which empathic emotion causes helping behavior. Empathic emotion causes helping behavior because it involves group identification, which motivates helping behavior toward other group members.

Based on a series of influential experiments, Batson (1991, 2011, 2018) defends “the empathy-altruism hypothesis (EAH)” according to which empathic emotion generates altruistic motivation. For example, when person X empathizes with person Y, who is suffering, X’s empathy causes X to be altruistically motivated to help Y. Batson’s experiments convincingly show that empathy causes helping behavior; for example, X’s empathizing with Y causes X to help Y (or at least causes X to be disposed to help Y). However, empathy-induced helping behavior is open to multiple interpretations other than EAH. There are many alternative interpretations, including the view that empathy-induced helping behavior is egoistically motivated rather than altruistically motivated.^1^ For instance, it is conceivable that X’s helping Y is due to X’s egoistic motivation to alleviate his own empathic distress [“the aversive-arousal reduction hypothesis (ARH)”]. X feels psychological distress when X empathizes with Y’s suffering. X helps Y because alleviating Y’s suffering is probably the best way to reduce X’s own empathic distress. X’s helping behavior is ultimately motivated by the egoistic concern for reducing X’s own empathic distress rather than by the altruistic concern for alleviating Y’s suffering.

This paper focuses on an account of empathy-induced helping behavior, which we call “the self-other merging hypothesis (SMH).” SMH can be formulated in different ways, but its basic idea is that X’s empathizing with Y causes a “merging” between X and Y. X helps Y because X is motivated by the concern for X’s own welfare and where, due to self-other merging, X’s “own welfare” includes Y’s welfare.

Whether one should accept EAH or SMH involves considering both empirical and theoretical issues, but this paper focuses on the latter. In particular, we focus on the interpretation of SMH. How should we interpret SMH? What does it mean to say that the “self” and the “other” are “merged”? Can there be an interpretation of SMH that is both conceptually coherent and psychologically plausible?

We will argue that the best interpretation of SMH appeals to the process of group identification (“Self-other Merging as Group Identification”); when empathizing with Y, X group identifies with Y, which motivates X to be helpful to Y. This interpretation of SMH (the “group identification interpretation”) is both (1) behaviorally adequate in the sense that it successfully predicts and explains the helping behavior exhibited in experimental settings and (2) is psychologically plausible in the sense that it does not attribute psychologically unrealistic beliefs, desires, etc. to X (e.g., the belief that X exists in two separate bodies).

We will then consider whether empathy-induced helping behavior is egoistic or altruistic (“Discussion: Is Empathy-Induced Helping Egoistic or Altruistic?”). Assuming (the group identification interpretation of) SMH, empathy-induced helping behavior does not adhere to the traditional dichotomy between egoism and altruism. We thus propose a new taxonomy according to which X’s act of helping Y is altruistic at the individual level [because X is motivated by X’s concern for Y’s third-person singular (his/her/their) welfare rather than X’s first-person singular (my) welfare] and egoistic at the group level [because X is motivated by X’s concern for Y’s welfare insofar as it is constitutive of X’s first-person plural (our) welfare].

## The Debate

### The Empathy-Altruism Hypothesis

According to Batson’s EAH, empathy or empathic concern is a possible source of altruistic motivation. X’s empathy for Y can make X altruistic toward Y. The two key concepts in EAH, “altruism” and “empathy,” are characterized as follows.

“Altruistic” behavior is defined as the one that is motivated by a state (e.g., a desire) “with the ultimate goal of increasing another’s welfare” (Batson, 2018, p. 22). For instance, X’s act of helping Y is altruistic if the act is motivated by X’s ultimate desire to increase the welfare of Y. To say that X’s desire to increase the welfare of Y is “ultimate” is to say that X desires the increased welfare of Y for its own sake, rather than it being instrumental in achieving some other goal, such as X’s selfish goal of feeling good that comes with helping Y.

“Empathy” or “empathic concern” is defined as an “other oriented emotion elicited by and congruent with the perceived welfare of someone in need” (Batson, 2018, p. 29).^2^ Here are some clarificatory notes on Batson’s definition. First, the “congruence” here is not necessarily the congruence of emotion (e.g., X feels the same emotion as Y) but rather of valence (e.g., both X and Y feel something negative). Second, “empathic concern” is an umbrella term that includes a range of other-oriented emotions for someone in need, such as sympathy, compassion, tenderness, soft-heartedness, sorrow, sadness, upset, distress, and grief. Third, the emotions under the umbrella of “empathic concern” are “other-oriented” in the sense that they involve feeling for the other (e.g., X’s sympathy for Y, X’s compassion for Y, and X’s sorry for Y).

Batson and his colleagues conducted a series of experiments to test EAH. In the “Katie Banks experiment” (Coke et al., 1978), for example, participants took a capsule (a placebo, unbeknown to them) and were either told that the capsule would have the effect of relaxing them (the relaxation side-effect condition) or that the capsule would have the effect of arousing them (the arousal side-effect condition). Participants then heard a news report on Katie Banks, who had been suffering and struggling after the tragic loss of her parents in an accident (this news report was, unbeknown to participants, entirely fictional). Participants were then either instructed to imagine how Katie felt about her situation (the imagine-her condition) or to observe the broadcasting techniques used in the news report (the observe condition). After hearing the news report, participants were presented with an opportunity to help Katie. The result was that participants in the relaxation side-effect/imagine-her condition were more likely to offer help to Katie than those in other conditions (the arousal side-effect/imagine-her condition, the relaxation side-effect/observe condition, and the arousal side-effect/observe condition). This suggests that the empathic imagining of Katie’s suffering increased the probability of helping behavior. Participants in the arousal side-effect/imagine-her condition were less likely to offer help, probably because their affective arousal (due to empathic imagining) was explained away as an effect of the capsule and hence it failed to be identified as empathic concern for Katie.

Thus, empathy does induce helping behavior. The crucial question is whether empathy-induced helping behavior is genuinely altruistic. According to EAH, empathy-induced helping behavior is genuinely altruistic. Participants in the relaxation side-effect/imagine-her condition offered help because empathizing with Katie induced the non-instrumental desire to help her. However, the behavior can also be explained as egoistic rather than altruistic. One such egoistic interpretation is provided by ARH: these participants exhibited helping behavior due to the egoistic desire to alleviate the negative emotion or distress caused by empathizing with Katie’s suffering. By empathizing with Katie’s suffering, participants felt a negative-valenced and distressful emotion, which motivated them reduce this emotion by helping to alleviate Katie’s suffering.

Note that there is an important asymmetry between egoistic hypotheses, such as ARH, and altruistic hypotheses, such as EAH. Altruistic hypotheses say that humans can be altruistically motivated; they do not rule out the possibility of egoistically motivated behavior. In contrast, egoistic hypotheses say that humans cannot be altruistically motivated; they do rule out the possibility of altruistically motivated behavior. Suppose that empathy-induced helping behavior in the Katie Banks experiment has a mixed set of motivations; some altruistic motivation to alleviate Katie’s suffering for its own sake and some egoistic motivation to alleviate one’s own empathic distress. This case of mixed motivation is coherent with altruistic hypotheses (because they do not rule out the possibility of egoistic motivation) but not with egoistic hypotheses (because they do rule out the possibility of altruistic motivation).

To see which hypothesis is correct, Batson and colleagues conducted the “Elaine experiment” (Batson et al., 1981). In this experiment, female participants observed a young woman named Elaine (who, unbeknown to participants, was a fictional person) through a computer monitor. Participants watched as Elaine received uncomfortable electric shocks. Participants were either informed that Elaine’s values and interests were very similar to their own (the similar-victim condition) or were very different (the dissimilar-victim condition). It turned out, however, that Elaine was especially sensitive to the shock, and participants were asked to help her by receiving the shock on her behalf. In one condition (the difficult-escape condition), participants were told that they had to stay in the experiment and watch Elaine receiving shocks if they did not volunteer to take her place. In the other condition (the easy-escape condition), they were told that they could leave if they did not volunteer. The result was that in the similar-victim condition, in which empathy is assumed to be elicited, ease of escape did not reduce the likelihood of the participant helping Elaine. This result contradicts ARH, according to which participants are motivated to reduce their own distress, and supports EAH, according to which participants are motivated to increase Elaine’s welfare. Ease of escape did reduce the likelihood of participants helping Elaine in the dissimilar-victim condition, which can be explained by the fact that participants in this condition had little altruistic motivation to help Elaine; they only had the egoistic motivation to get out of a rather uncomfortable experiment.

This is, however, not a conclusive refutation of ARH. For example, there is still room to argue that participants did not escape because they cared about Elaine’s well-being, but rather because they believed that a physical escape would not bring a psychological escape; leaving the experiment would not alleviate their empathic distress. But this idea is also challenged by the experiments conducted by Stocks et al. (2009), which suggests that empathy promotes helping behavior even when a psychological escape is available.

### The Self-Other Merging Hypothesis

Although EAH has been supported by several studies, it is still a controversial hypothesis. Our focus in this paper is the debate on EAH and SMH.^3^ SMH can be formulated in different ways, but its basic idea is that X’s empathizing with Y causes a “merging” between X and Y. X helps Y because X is motivated by the concern for X’s own welfare and where, due to self-other merging, X’s “own welfare” includes Y’s welfare.

Among the proponents of SMH,^4^ we focus on Cialdini et al. (1997) whose work plays the central role in the debate on EAH and SMH. Cialdini et al. (1997) conducted a series of experiments whose results suggest that the real cause of altruistic behavior is “oneness” or “self-other merging.”

In one of their experiments, the participants (introductory psychology students, including both males and females, at Arizona State University) were assigned to one of the conditions below and were asked to imagine a person who is associated with the assigned condition:

1. The near-stranger condition: “a man/woman you do not really know…someone you would recognize from class, but not say ‘hello’ to if you passed each other on campus.”
2. The acquaintance condition: “a man/woman who you do not know really well, but you would stop and chat with him/her for a few minutes if you passed each other on campus.”
3. The good friend condition: “a man/woman who is a friend of yours, who you sometimes go out with outside of school.”
4. The family member condition: “your closest male/female family member, a sibling if possible.”

All participants were asked to think about a situation where the person they imagined had been evicted from their apartment. Then, they indicated to what extent they wanted to help the imagined person, choosing from seven options, ranging from being totally unhelpful (i.e., doing nothing) to being extremely helpful (i.e., offering to let the imagined person live with the participant rent-free). After that, participants indicated the extent of “oneness” with the imagined person on a seven-point scale, ranging from “not at all” to “extremely.” In addition, Cialdini and colleagues used the inclusion of other in self scale to evaluate participants’ feelings of “oneness”.^5^ The participants also indicated their empathic concern, sadness, and personal distress on a seven-point scale.

The result suggests that closeness of relationship is correlated with greater empathic concern as well as greater oneness and that empathic concern and oneness are predictive of helping behavior. Crucially, when oneness is statistically controlled, empathic concern is not predictive of helping. In contrast, when empathic concern is controlled, oneness is still predictive of helping behavior. Cialdini and colleagues take this result to show that the primary cause of helping behavior is “oneness” or “self-other merging” and that empathy plays only a mediatory role: “Upon experiencing empathic concern for another, then, an individual is consequently informed of a likely degree of oneness with that other, and prosocial action is more probable as a result” (Cialdini et al., 1997, p. 491).

If the self-other merging is the real cause of helping behavior, then, it becomes less clear whether the helping behavior exhibited in experimental settings is truly altruistic. Cialdini and colleagues thus state that SMH “seriously undermines the logic of the empathy-altruism hypothesis” by compromising “the distinction between selflessness and selfishness” (Cialdini et al., 1997, p. 481). Note that Cialdini and colleagues do not claim that the helping behavior exhibited in experimental settings is egoistic. It is not altruistic, nor egoistic, but *nonaltruistic*. SMH, according to Cialdini and colleagues, goes beyond the traditional dichotomy between egoism and altruism.

## Self-Other Merging As Group Identification

### May’s Challenge

Batson responded to Cialdini and colleagues by conducting experiments that cast doubt on SMH (Batson et al., 1997) and by indicating the methodological problems present in the experiments by Cialdini and colleagues (Batson, 2018, Chapter 10). These empirical issues about SMH, however, are outside the scope of this paper. Our focus is rather on the theoretical issues surrounding SMH, particularly the theoretical issue of how to interpret SMH. How should we interpret SMH? What does it mean to say that the “self” and the “other” are “merged”? Can there be an interpretation of SMH that is both conceptually coherent and psychologically plausible?

May (2011, 2018) examines three possible interpretations of SMH and argues that all face serious difficulties. According to the first interpretation (let us call it “the peculiar belief interpretation”), when X empathizes with Y, X believes that they exist simultaneously in two separate bodies (i.e., in the body of X and in the body of Y). The peculiar belief interpretation is problematic because it is psychologically unrealistic that people have such a wildly implausible belief when they empathize with others. Cialdini and colleagues themselves also seem to deny this possibility when they write: “What is merged is conceptual, not physical. We are not suggesting that individuals with overlapping identities confuse their physical beings or situations with those of the other” (Cialdini et al., 1997, p. 482). According to the second interpretation (“the indeterminate identity interpretation”), when X empathizes with Y, the personal identities of X and Y become indeterminate. The indeterminate identity interpretation is problematic because it is empirically unclear whether humans have the psychological ability to represent indeterminate identities of persons. Another problem, according to May, is that the indeterminate identity interpretation cannot explain the helping behavior exhibited in experimental settings because X’s act of helping Y is possible only if X represents Y to be another person, distinct from oneself. According to the third interpretation (“the property interpretation”), when X empathizes with Y, X believes that some of X’s aspects or properties are in Y’s body (while X and Y are represented as distinct persons). The property interpretation faces the same problem as the peculiar belief interpretation: it is psychologically unrealistic that X believes that some of X’s aspects or properties are in Y’s body. May also points out that even if we accept that X holds such a belief, we still need to attribute some altruistic motivation to X in order to account for X helping Y. It is not clear why X, non-altruistically motivated, helps Y just because X’s properties or aspects are in Y’s body.

May thus concludes that SMH is in serious trouble: “the self-other merging explanation fails to explain the empathy-helping relationship on conceptual grounds, regardless of the experiments Cialdini et al. (1997) report” (May, 2011, p. 26).

### The Group Identification Interpretation

May’s challenge is important not because it is a conclusive objection to SMH (arguably, May’s reading of Cialdini and colleagues is not very charitable) but rather because it is useful for clarifying what needs to be done in order to defend SMH. Generalizing May’s challenge, a plausible interpretation of SMH needs to meet the following conditions:

1. It must be behaviorally adequate in the sense that SMH, thus interpreted, successfully predicts and explains the helping behavior exhibited in experimental settings.
2. It must be psychologically plausible in the sense that SMH, thus interpreted, does not posit psychologically unrealistic beliefs, desires, etc.

May’s objection to the peculiar belief interpretation is that it is not psychologically plausible; it attributes psychologically unrealistic beliefs to people. It is psychologically unrealistic, for example, that a participant in the Elaine experiment – let us call him “Reynie” – believes that he exists in two separate bodies. One of May’s objections to the indeterminate identity interpretation is that it is not behaviorally adequate; it fails to explain helping behavior. Reynie’s act of helping Elaine is possible only if Reynie represents Elaine as another person, distinct from himself.

We will argue that there is a plausible interpretation of SMH, which is very likely to be behaviorally adequate and psychologically plausible. This interpretation, which we call “the group identification interpretation,” understands self-other merging as the process in which self and other merge into *one* group. More precisely, self-other merging involves group identification, where group identification is understood as the process in which one achieves a form of self-conception as a group member.^6^ One’s self-conception as a group member manifests in using the first-person plural (we) referring to oneself and other group members; for example, one’s self-conception as a “Harvard dad” manifests in using the first-person plural (we) when referring to oneself and others in the Harvard community (e.g., in the context of the Harvard-Yale football game). It has been suggested in social psychology and philosophy that group identification plays a crucial role in collective, cooperative, and collaborative behaviors (Turner, 1982; Brewer, 1991; Pacherie, 2013; Salice and Miyazono, 2020); a person’s self-conception as a Harvard dad can, for example, facilitate collective, cooperative, and collaborative behaviors with others in the Harvard community.

The group identification interpretation of SMH amounts to the following: X’s empathy-induced helping behavior toward Y is explained by the fact that when X empathizes with Y, X group identifies with Y and thereby comes to conceive of Y’s welfare as being constitutive of X’s first-person plural (our) welfare. Some clarifications are in order.

First, the group identification interpretation of SMH provides us with an account of empathy-induced helping behavior (e.g., Reynie’s empathy-induced helping behavior toward Elaine) rather than helping behavior in general. It is compatible with the possibility that helping behavior in some cases is not caused by empathy and has nothing to do with group identification.

Second, according to the group identification interpretation, when empathy causes helping behavior (e.g., Reynie’s empathy with Elaine causes him to help her), it does so because of group identification (e.g., Reynie’s group identification with Elaine). It is compatible with the possibility that empathy does not always cause helping behavior, or that group identification does not always cause helping behavior.

Third, the group identification interpretation is not committed to the idea that group identification is necessarily associated with empathy. It is compatible with the possibility that group identification happens without empathy in some cases, or that empathy-independent group identification causes helping behavior in some cases (see our distinction between “empathy-induced, group identification-driven helping behavior” and “group identification-driven helping behavior” in “The Traditional Dichotomy”).

Fourth, the group identification interpretation is compatible with different theories of group and social identity. It is theoretically neutral on what groups are and how they work; for example, whether groups are grounded in common features (such as common values and interests between Elaine and Reynie) or shared activities (such as emotional sharing between Elaine and Reynie).

Let us now closely examine the group identification interpretation. The group identification interpretation is psychologically plausible, which is especially clear when we compare the group identification interpretation with the peculiar belief interpretation. The peculiar belief interpretation is not psychologically plausible because it is psychologically unrealistic that Reynie believes that he exists in two separate bodies. The group identification interpretation is different from the peculiar belief interpretation for two reasons. The first reason relates to the content of group identification. The group identification interpretation does not attribute a belief with psychologically unrealistic content – such as the content that he exists in two separate bodies – to Reynie. The group identification interpretation involves the idea that Reynie and Elaine share a social identity, but it does not involve the idea that Reynie exists in two separate bodies (in his body and in Elaine’s body).

The second reason relates to the attitude of group identification. The group identification interpretation does not attribute a belief to Reynie in the first place. Salice and Miyazono (2020) propose a non-doxastic account of group identification, according to which group identification is cashed out in terms of non-doxastic representations. They reject the idea that group identification is a doxastic process in which, for example, X comes to believe that X is a member of a particular group; such a belief does not account for the motivational force of group identification. Rather, group identification involves a “pushmi-pullyu representation” (Millikan, 1995, 2004): when X group identifies, X forms a representation with both descriptive content (e.g., describing oneself as a member of a group) as well as directive content (e.g., directing oneself to behave as a group member). Generally, pushmi-pullyu representations are representational states that are evolutionarily and structurally primitive. Pushmi-pullyu representations have both descriptive content and directive content, while beliefs have descriptive content only. Pushmi-pullyu representations are intrinsically motivating without being combined with a conative state, while beliefs are not intrinsically motivating.

The group identification interpretation is behaviorally adequate. Experimental studies, in particular the ones in the minimal group paradigm (for an overview, see Diehl, 1990), show that group identification motivates pro-group behavior. In a classic study by Tajfel et al. (1971), for example, a person with the self-conception as a Kandinsky-lover (as opposed to a Klee-lover) was motivated to be helpful to other Kandinsky-lovers (as opposed to Klee-lovers). In the experiment, participants first indicated their aesthetic preferences in response to given pairs of paintings. Based on their preferences, they were told that they were either Kandinsky-lovers or Klee-lovers (while, unbeknownst to them, they were randomly assigned to either the Kandinsky-lover group or the Klee-lover group). In the next task, participants were asked to distribute real monetary rewards to other participants. During this task, in-group favoritism was observed: Kandinsky-lovers favored other Kandinsky-lovers and Klee-lovers favored other Klee-lovers.

The group identification interpretation is theoretically plausible because it enables a unified and parsimonious account of helping behavior in both the minimal group studies by Tajfel and colleagues and the empathy-altruism studies by Batson and colleagues.^7^ Other things being equal, it is better to have a single and unified account of helping behavior in two sets of studies rather than to have two different accounts. Helping behavior in both sets of studies is driven by the process of group identification. In the Klee-Kandinsky experiment, for example, a participant, let us call him “Sticky,” self-identifies as a Kandinsky-lover, which motivates him to be helpful to other Kandinsky-lovers. Similarly, when Reynie empathizes with Elaine in the Elaine experiment, he group identifies with Elaine, which motivates him to be helpful to Elaine.

The group identification interpretation is also empirically plausible because there are in fact important similarities between how these two sets of studies were conducted and what the participants were asked to do in them. First, participants in both the Klee-Kandinsky experiment and the Elaine experiment went through similar processes: They were, for instance, informed of the similarities between themselves and a person and then offered an opportunity to be helpful to the person. Sticky, in the Klee-Kandinsky experiment, was first informed of other Kandinsky-lovers and was then offered the opportunity to be helpful to them. Reynie, in the Elaine experiment, was first informed of the similarities between himself and Elaine and was then offered the opportunity to be helpful to Elaine. Second, the responses of participants were similar: for instance, they both helped a person. Sticky was helpful to other Kandinsky-lovers and Reynie was helpful to Elaine. These remarkable structural similarities between the Klee-Kandinsky experiment and the Elaine experiment make it empirically plausible that they share the same psychological explanation, which is exactly what the group identification interpretation offers.

### Empathy and Self-Conscious Emotions

According to the group identification interpretation of SMH, when X empathizes with Y, X group identifies with Y. There are at least two different interpretations of how empathy leads to group identification. According to what we call “the causal interpretation,” the relationship between empathy and group identification is causal; group identification is caused by empathy. X’s empathizing with Y causes X to group identify with Y because, for example, empathy highlights some commonalities between X and Y, which causally trigger the process of group identification.^8^ In contrast, according to what we call “the constitutive interpretation,” the relationship between empathy and group identification is constitutive; group identification is constitutive of empathy. It is not the case that first X empathizes with Y, which then causes X to group identify with Y. Rather, X’s empathizing with Y already involves X group identifying with Y. In other words, it is part of X’s empathizing with Y that X group identifies with Y and conceives Y to be a constitutive part of X’s social self.

Both interpretations are coherent and, for the purpose of this paper, we are neutral on this issue. Arguably, however, the constitutive interpretation is more interesting than the causal interpretation from a philosophical point of view. In this context, it is useful to compare empathy with self-conscious emotions, such as pride or shame. Salice and Montes Sánchez (2016) argue that other-induced self-conscious emotion is based on group identification. For instance, when a father is proud of his daughter for winning a Nobel Prize, the father group identifies with his daughter: “When you feel proud of your daughter, the emotion is still about yourself, your self, but this is about your self insofar as it is your social self. Seeing yourself as a member of a group, the actions and/or achievements of the other members acquire relevance when it comes to assessing your social self, and this is what triggers the emotive response” (Salice and Montes Sánchez, 2016, p. 7). Linguistically, it is natural to say that “the father is proud of his daughter,” which seems to indicate that the father’s pride is about his daughter as opposed to the father himself, but Salice and Montes Sánchez think that the linguistic expression does not reveal what the pride is really about. They distinguish the “target” of self-conscious emotions from their “focus”; the daughter and her achievement are the focus of the father’s pride (and that is what the linguistic expression “the father is proud of his daughter” reveals), but the pride is about the father’s social self, which is the target of the pride.

This proposal provides us with a plausible solution to a puzzle concerning two ideas that are independently plausible but incongruous with one another. The first idea is that self-conscious emotions are essentially first-personal (e.g., pride is essentially about oneself) and the second idea is that self-conscious emotions can be induced by what somebody else does (e.g., a father can be proud of what his daughter has achieved). These two ideas are in fact compatible with each other, according to Salice and Montes Sánchez. It is certainly true that a father can be proud of his daughter for winning a Nobel Prize (the second idea), but the father’s pride is not merely about his daughter; the father pride is rather about his daughter as a constitutive part of the father’s social self (the first idea).

Salice and Montes Sánchez’s proposal might be applied to empathy as well. Batson’s definition of empathy or empathic concern as an “other-oriented emotion” seems to suggest that when X empathizes with Y, X’s empathic emotion is about Y rather than X. But there is an alternative account of empathy, which is analogous to Salice and Montes Sánchez’s account of self-conscious emotions: when X empathizes with Y, X’s empathy is not merely about Y; X’s empathy for Y is about Y as a constitutive part of X’s social self. When Reynie empathizes with Elaine in the Elaine experiment, for example, Reynie’s empathy is not merely about Elaine; rather Reynie’s empathy is about Elaine as a constitutive part of Reynie’s social self. Linguistically, it is natural to say that “Reynie empathizes with Elaine,” which seems to indicate that Reynie’s empathy is about Elaine as opposed to Reynie himself. But perhaps the linguistic expression does not reveal what empathy is really about. Following Salice and Montes Sánchez, we might distinguish the “target” of empathy from its “focus”; Elaine and her suffering are the focus of Reynie’s empathy, but the empathy is about Reynie’s social self, which is the target of his empathy.

This proposal is coherent with the constitutive interpretation according to which group identification is constitutive of empathy. It is not the case that Reynie first empathizes with Elaine, which then causes him to group identify with Elaine. Rather, Reynie’s empathizing with Elaine already involves him group identifying with Elaine. In other words, it is part of Reynie’s empathizing with Elaine that Reynie group identifies with Elaine and conceives Elaine to be a constitutive part of his social self.

### Objections and Responses

We will now discuss two possible objections to the group identification interpretation.

The first objection might be that group identification typically happens when the group in question has some desirable characteristics. Identifying oneself with a positive and desirable group can bring some psychological benefits, such as enhanced self-esteem. For example, by identifying himself as a “Harvard dad,” a father’s self-esteem can be enhanced. However, if this is how group identification works in general, then it is hard to see how group identification can happen in the context of Batson’s empathy-altruism studies. Unlike the Harvard community, the group of Reynie and Elaine does not seem to have any particularly desirable characteristics. Unlike group identifying as a “Harvard dad,” group identifying with Elaine does not enhance Reynie’s self-esteem at all. On the contrary, such group identification might threaten Reynie’s self-esteem given Elaine’s undesirable situation (Elaine is, after all, in an undesirable position where she is at risk of suffering from electrical shocks).

Our response to this objection is that group identification is not intrinsically related to self-esteem. The role of self-esteem in group identification has been studied and discussed (Rubin and Hewstone, 1998; Hewstone et al., 2002), but it is clear that self-esteem does not explain everything about group identification. In particular, self-esteem does not seem to explain group identification in the minimal group experiments. For example, Tajfel et al. (1971) observed that group identification as an over-estimator (i.e., someone who over-estimates the number of dots on a screen) or under-estimator (i.e., someone who under-estimates the number of dots on a screen) caused in-group favoritism; over-estimators favored other over-estimators and under-estimators favored other under-estimators. It is difficult to see how group identification as an over-estimator or an under-estimator can enhance one’s self-esteem. The minimal group experiments seem to show that group identification can be driven by similarities (e.g., the person is an over-estimator just like you) rather than desirability (e.g., it is desirable to be an over-estimator).

Relatedly, Salice and Montes Sánchez (2016) reject the idea that group identification is motivated by a concern for self-esteem. The self-esteem hypothesis is certainly plausible as an account of a father’s pride in his daughter’s accomplishments. The father’s pride is based on him group identifying with his daughter, which does seem to enhance his self-esteem. But the self-esteem hypothesis is implausible as an account of a father feeling ashamed of his daughter’s actions (e.g., her mistakes and crimes). The father’s shame is, according to Salice and Montes Sánchez, based on him group identifying with his daughter, which does not enhance his self-esteem at all. The father’s self-esteem is threatened, rather than enhanced, when he group identifies with her.

The second objection might be that group identification cannot explain empathy-induced helping behavior because the latter has a broader scope than the former. X’s empathy can extend beyond the boundary of X’s own group. X can empathize with Y, and can be motivated to be helpful to Y, even though X and Y do not share a psychologically significant group (e.g., school, workplace, hometown, nationality, or ethnic origin). For example, we can empathize with a woman who is trapped in a building on fire and can be motivated to go into the building and save her life, even though she is a complete stranger to us. Or, we can empathize with suffering children in Africa and can be motivated to donate money to alleviate their suffering, even though we do not know anything about who they are. These cases appear to be the counterexamples for the group identification interpretation which explains empathy-induced helping behavior in terms of group identification.

This is certainly an important objection to our proposal, but there are several possible responses. The responses can be divided into two groups. First, we might insist that group identification does occur even in these difficult cases; for example, we actually group identify with suffering children in Africa. These cases are not counterexamples because they do involve group identification. Second, we might deny the assumption that the helping behavior in these difficult cases is caused by empathy; for example, it is not the case that empathy causes us to go into the building on fire to save the woman’s life. These cases are not counterexamples because they have nothing to do with empathy-induced helping behavior.

Let us start with the first response. Let us grant, for the sake of argument, that we do not share a psychologically significant group with the suffering children in Africa. However, this does not rule out the possibility that we group identify with the African children. In general, X’s group identification with Y does not presuppose that X and Y share a psychologically significant group. Group identification can be driven by rather trivial groups, which was nicely demonstrated in the minimal group studies. Trivial groups that are instantly created, such as the “Kandinsky-lovers” or “over-estimators” groups, can trigger group identification and motivate in-group helping behavior. In the Elaine experiment, Reynie was informed of the similarities between himself and Elaine. It is not surprising at all that the informed similarities were enough for Reynie to group identify with Elaine.

How does group identification with the African children work, exactly? In the present case, we do not even know whether the children are Kandinsky-lovers or Klee-lovers, whether they are over-estimators or under-estimators, etc. A suggestion would be that group identification in this case is grounded in empathic affective mirroring.^9^ We put ourselves into the shoes of a suffering child in Africa and empathically share her suffering. The shared experience of suffering creates a salient similarity between the child and us, which facilitates our group identification with her.

Let us move on to the second response. As we already noted, the group identification interpretation is an account of empathy-induced helping behavior rather than helping behavior in general. It does not say anything about the helping behavior that is not caused by empathy. Now, it is possible that the act of rescuing the woman in the building on fire is not caused by empathy in the first place. In fact, saving a person’s life in the context of an emergency does not seem to require empathy. As Bloom notes, we can be motivated to save a child from drowning without empathizing with the child: “You do not need empathy to realize that it’s wrong to let a child drown. Any normal person would just wade in and scoop up the child, without bothering with any of this empathic hoo-ha” (Bloom, 2016, p. 22). The helping behavior within a group boundary is explained by empathy, while the helping behavior beyond a group boundary (e.g., helping African children who we know almost nothing about, helping a stranger in the building on fire, etc.) might be explained by reason or domain-general reasoning processes. Again, Bloom notes: “it is reason that leads us to recognize, despite what our feelings tell us, that a child in a faraway land matters as much as our neighbor’s child” (Bloom, 2016, p. 51).

Of course, it is possible to modify the case in such a way that the act of rescuing the woman in the building on fire is likely to be caused by empathy. However, with such a revision, this case becomes less problematic for the group identification interpretation. For instance, we might modify the case in such a way that we are very likely to empathize with the woman in the building on fire because of the vivid memory of our own experience of being trapped in a building on fire. However, this revised case might not be very problematic for the group identification interpretation; we can easily group identify with the woman in this case because of the salient similarity between the woman and us; that is, the shared experience of being trapped in a building on fire.

## Discussion: Is Empathy-Induced Helping Egoistic or Altruistic?

### The Traditional Dichotomy

The rest of this paper will discuss whether empathy-induced helping behavior is egoistic or altruistic in light of the group identification interpretation of SMH. If the group identification interpretation of SMH is correct, is empathy-induced helping behavior egoistic or altruistic?

According to the group identification interpretation of SMH, empathy-induced helping behavior, such as the behavior exhibited in Batson’s experiments, is driven by group identification. For example, when Reynie empathizes with Elaine, he group identifies with her, which drives him to help her. Let us call such helping behavior “empathy-induced, group identification-driven helping behavior” (EGHB). EGHB is a form of what we call “group identification-driven helping behavior” (GHB). GHB can be, but does not have to be, induced by empathy. If it is induced by empathy, then it counts as EGHB. If it is not induced by empathy, then it does not count as EGHB. For instance, Reynie’s helping behavior toward Elaine is EGHB, while Sticky’s helping behavior toward other Kandinsky-lovers is GHB but probably not EGHB.

EGHB does not comfortably fit in the traditional category of egoistic behavior. EGHB seems to be different from purely egoistic helping behavior in which X helping Y is motivated by X’s concern for X’s first-person singular (my) welfare (e.g., alleviating *my* empathic distress). X’s EGHB toward Y seems to be motivated by something beyond X’s first-person singular welfare. For example, Reynie’s EGHB in the Elaine experiment seems to be motivated by something other than Reynie’s concern for his first-person singular welfare.

This observation can be challenged. A challenge is that group identification is, in general, motivated by the egoistic concern for one’s own self-esteem; group identification aims to enhance one’s own self-esteem by identifying oneself with a group that has some desirable characteristics. However, as previously argued, the self-esteem hypothesis cannot be the full explanation of group identification. The self-esteem account is especially problematic in the kind of cases we are interested in because Reynie group identifying with Elaine does not seem to enhance Reynie’s self-esteem. Another problem is that even if the group identification itself is egoistically motivated, it does not automatically follow from this that EGHB is also egoistically motivated. It is at least conceivable that, on the one hand, the act of conceiving of oneself as a group member is motivated by the egoistic goal of enhancing one’s own self-esteem, while, on the other hand, the act of helping other group members is not motivated by the same egoistic goal.

Another challenge is that EGHB is motivated by the egoistic expectation of reciprocity, such as the egoistic expectation that group members who you have helped will also help you in the future. But there are some difficulties with this proposal. For example, EGHB can happen even when there is almost no chance of reciprocity (e.g., empathy-induced monetary donations to alleviate the suffering of children in Africa). Still, some might insist that the reciprocity account can be defended from an evolutionary perspective; the evolutionary role of EGHB might be, for example, to facilitate reciprocal helping in groups. We do not rule out such a possibility, but it has little to do with our discussion of egoism and altruism. Biological aims or purposes need to be carefully distinguished from a person’s aims or purposes. It could be argued that the biological aim of the psychological mechanisms for EGHB is to facilitate reciprocal helping and that these psychological mechanisms have been selected for their contribution to reciprocal helping. But this does not imply that Reynie is personally motivated by the egoistic expectation that Elaine will help him in the future. As Batson points out, the evolutionary reciprocity account “says nothing about whether we ever seek to promote another’s welfare for his or her sake rather than our own” (Batson, 2018, p. 17).

Thus, EGHB does not fit nicely into the traditional category of egoistic behavior. But EGHB does not fit comfortably into the traditional category of altruistic behavior either. EGHB seems to be different from purely altruistic helping behavior in which X helping Y is motivated by X’s concern for Y’s third-person singular (his/her/their) welfare (e.g., alleviating *her* suffering), which is conceived to be distinct from X’s welfare. X’s EGHB toward Y seems to be motivated by X’s concern for Y’s third-person singular welfare insofar as Y’s third-person singular welfare is constitutive of X’s first-person plural (our) welfare. For example, Reynie’s EGHB toward Elaine seems to be motivated by Reynie’s concern for Elaine’s third-person singular welfare insofar as Elaine’s third-person singular welfare is constitutive of Reynie’s first-person plural (our) welfare.

One might think is that Reynie’s concern for Elaine’s third-person singular welfare is only instrumental to his concern for his first-person plural welfare. Reynie does not have a non-instrumental concern for Elaine’s welfare, which suggests that Reynie’s EGHB toward Elaine is not altruistic in any interesting sense – it is simply egoistic after all. Reynie’s EGHB toward Elaine is analogous to the egoistic helping behavior where X’s concern for Y’s third-person singular welfare is only instrumental to X’s egoistic motivation for alleviating X’s own empathic distress. In both cases, the concern for the third-person singular welfare is only instrumental to the ultimate concern for first-person (singular or plural) welfare.

However, we resist the interpretation that Reynie’s concern for Elaine’s third-person singular welfare is only instrumental to his concern for his own first-person plural welfare. There is a crucial disanalogy between Reynie’s EGHB toward Elaine and X’s egoistic helping behavior toward Y for the sake of alleviating X’s own empathic distress. In the latter case, X’s concern for Y’s third-person singular welfare is only instrumental to X’s concern for X’s first-person singular welfare. This is due to the fact that X’s first-person singular welfare and Y’s third-person singular welfare are ontologically independent of each other; X’s first-person singular welfare is causally influenced by, but not constituted by, Y’s third-person singular welfare. In the former case, in contrast, Reynie’s first-person plural welfare is constituted by, but is not causally influenced by, Elaine’s third-person singular welfare. Reynie’s first-person plural welfare and Elaine’s third-person singular welfare are not ontologically independent from one another – the latter is a constitutive part of the former. But then it would be inappropriate to say that Reynie’s concern for Elaine’s third-person singular welfare is only instrumental to Reynie’s concern for his first-person plural welfare. Reynie’s concern for Elaine’s third-person singular welfare is not distinct from his concern for his first-person plural welfare. It is part of having concern for his first-person plural welfare that Reynie has (non-instrumental) concern for Elaine’s third-person singular welfare. In general, it is part of having concern for Y that one has (non-instrumental) concern for X, when X is constitutive of Y.

### A New Taxonomy

It is difficult to locate EGHB in the traditional dichotomy between egoism and altruism. Should we conclude, then, that EGHB is neither egoistic nor altruistic? This is certainly a plausible option for some actions, in particular those that appear to have nothing to do with anybody’s welfare, such as the act of working on a great painting for its own sake rather than for one’s own reputation as a painter. But this option is implausible for EGHB, which is obviously related to the motivation for increasing somebody’s welfare.

The discussions above suggest that it is difficult to locate EGHB in the traditional egoism/altruism dichotomy not because our understanding of EGHB is insufficient but rather because the traditional egoism/altruism dichotomy is inadequate. It is inadequate because the possibility of EGHB (and GHB in general) is not taken into account in the traditional dichotomy in the first place. Cialdini and colleagues make a similar point when they say that SMH goes beyond “the distinction between selflessness and selfishness” (Cialdini et al., 1997, p. 482).

This problem does not just concern EGHB, it also concerns GHB in general. For example, let us consider the following example by Salice and Satne:

Imagine that Alba and Simon are members of a small alpine community. Recently, there was discussion within the community about the idea of building a small bridge over the adjacent brook in order to reach the next village down in the valley. In contrast to Simon, Alba does not have a desire to build a new bridge, but she is not opposed to it either. Eventually, the community convenes to make a decision. Alba cannot attend the meeting, but she is informed afterward that the group decided to build the bridge, in so doing they have also distributed the responsibilities regarding who will be doing what: for instance, Alba is to produce the list of necessary materials to build the bridge and Simon is involved in seeking these materials from providers. […] Alba is not averse to the idea of the bridge (nor is she in favor of it) and she usually takes care of the kind of duties that the community has assigned to her. The only thing that matters for her is that the community decided in favor of building the bridge, which can be phrased as (from Alba’s perspective): we intend to build the bridge (Salice and Satne, 2020, p. 615).

In this case, Alba and Simon cooperate in such a way that Alba helps Simon to find the necessary materials. Alba’s act of helping Simon can reasonably be understood as an example of GHB, although it is probably not induced by empathizing – it is probably not EGHB. Is Alba’s GHB egoistic or altruistic? Salice and Satne remain neutral on this question, which is probably (partly) due to the difficulty of locating GHB in the traditional egoism/altruism dichotomy. On the one hand, Alba’s GHB toward Simon seems to be different from a purely egoistic helping behavior where Alba’s act of helping Simon is motivated by Alba’s concern for her own first-person singular (my) welfare (e.g., achieving *my* good reputation). Alba’s GHB toward Simon seems to be motivated by something other than Alba’s concern for her first-person singular welfare. On the other hand, Alba’s GHB toward Simon seems to be different from a purely altruistic helping behavior where Alba’s act of helping Simon is motivated by her concern for Simon’s third-person singular (his) welfare (e.g., solving *his* problems), which is conceived to be independent of Alba’s welfare. Alba’s GHB toward Simon seems to be motivated by her concern for Simon’s third-person singular welfare insofar as Simon’s third-person singular welfare is constitutive of Alba’s first-person plural (our) welfare.

What we need, then, is a new taxonomy in which EGHB (and GHB in general) is taken into account. We propose to distinguish the egoistic/altruistic distinction at the individual level from the egoistic/altruistic distinction at the group level such that EGHB (and GHB in general) can be regarded as altruistic at the individual level and as egoistic at the group level simultaneously. Reynie’s EGHB toward Elaine is motivated by his concern for Elaine’s welfare. Elaine’s welfare, which is the target of Reynie’s concern, is third-person singular (her) welfare rather than first-person singular (my) welfare at the individual level, which is why Reynie’s EGHB is altruistic at the individual level, while Elaine’s welfare is constitutive of first-person plural (our) welfare rather than third-person plural (their) welfare at the group level, which is why Reynie’s EGHB is egoistic at the group level.

EGHB is altruistic at the individual level in the sense that, for example, Reynie’s EGHB toward Elaine is motivated by Reynie’s concern for Elaine’s third-person singular (her)^10^ welfare rather than Reynie’s first-person singular (my) welfare at the individual level. This explains why EGHB is different from purely egoistic helping behavior in which X helping Y is motivated by X’s concern for X’s first-person singular (my) welfare (e.g., alleviating *my* psychological distress). Purely egoistic helping behavior is egoistic at the individual level in the sense that it is motivated by the concern for first-person singular (my) welfare, while EGHB is altruistic at the individual level in the sense that it is motivated by the concern for third-person singular (his/her/their) welfare.

At the same time, EGHB is egoistic at the group level in the sense that, for example, Reynie’s EGHB toward Elaine is motivated by Reynie’s concern for Elaine’s welfare in so far as Elaine’s welfare is constitutive of Reynie’s first-person plural (our) welfare at the group level.^11^ This explains why EGHB is different from purely altruistic helping behavior in which X helping Y is motivated by X’s concern for Y’s third-person singular (his/her/their) welfare (e.g., alleviating her suffering), which is conceived to be independent of X’s welfare. Both purely altruistic helping behavior and EGHB are altruistic at the individual level in the sense that they are motivated by the concern for third-person singular (his/her/their) welfare rather than first-person singular (my) welfare. Unlike purely altruistic helping behavior, however, EGHB is egoistic at the group level in the sense that it is motivated by third-person singular welfare insofar as it is constitutive of first-person plural (our) welfare rather than third-person plural (their) welfare.

Thus, we have reached an answer to our question as to whether the empathy-induced helping behavior is egoistic or altruistic according to the group identification interpretation of SMH. Our answer is that it is both egoistic and altruistic. More precisely, it is altruistic at the individual level and egoistic at the group level.

## Conclusion

Our central idea was that group identification is the key to understanding the process in which empathy motivates helping behavior. Empathy motivates helping behavior because it involves group identification, which motivates helping behavior toward other group members.

Our focus was on SMH according to which empathy-induced helping behavior is due to the “merging” between the helping agent and the helped agent. We argued that SMH should be interpreted in terms of group identification. The group identification interpretation of SMH is both behaviorally adequate (i.e., successfully predicts and explains the helping behavior in the experimental settings) and psychologically plausible (i.e., does not posit psychologically unrealistic beliefs and desires; “Self-other Merging as Group Identification”) Empathy-induced helping behavior, according to the group identification interpretation of the SMH, does not fit comfortably into the traditional egoism/altruism dichotomy. We thus proposed a new taxonomy according to which empathy-induced helping behavior is both altruistic at the individual level and egoistic at the group level (“Discussion: Is Empathy-Induced Helping Egoistic or Altruistic?”).

## Data Availability Statement

The original contributions presented in the study are included in the article/supplementary material, further inquiries can be directed to the corresponding author.

## Author Contributions

All authors listed, have made substantial, direct and intellectual contribution to the work, and approved it for publication.

## Funding

KM acknowledges the support of JSPS KAKENHI (grant number 21H00464).

## Conflict of Interest

The authors declare that the research was conducted in the absence of any commercial or financial relationships that could be construed as a potential conflict of interest.

The handling Editor declared a past co-authorship with one of the author KM.

## Publisher’s Note

All claims expressed in this article are solely those of the authors and do not necessarily represent those of their affiliated organizations, or those of the publisher, the editors and the reviewers. Any product that may be evaluated in this article, or claim that may be made by its manufacturer, is not guaranteed or endorsed by the publisher.

## Footnotes

^1^Useful literature reviews include Stich et al. (2010), Doris et al. (2020), and Miyazono and Bortolotti (2021, Chapter 5).

^2^Batson (2018) carefully distinguishes “empathic concern” from other psychological states and traits called “empathy,” including: (1) knowing another person’s thoughts and feelings; (2) feeling as another feels; (3) imagining how another feels; (4) imagining how you would feel in another’s place; (4) feeling self-oriented distress (including anxiety or unease) at witnessing another’s suffering; and (5) a general disposition, or trait, to feel for others.

^3^SMH is often taken to be an alternative to EAH, but the relationship between SMH and EAH is complex, or so we argue. As we will see in “Discussion: Is Empathy-Induced Helping Egoistic or Altruistic?”, our interpretation of SMH (the group identification interpretation) implies that EAH is correct at least in the individual level (but not in the group level); that is, the empathy-induced helping behavior is altruistic at the individual level (but not in the group level). See “Discussion: Is Empathy-Induced Helping Egoistic or Altruistic?” for details.

^4^Batson (2018) distinguishes four versions of SMH. According to the first version, “Self-Other Identification,” when X empathizes with Y, X “identifies” with Y (Hornstein, 1978; Lerner, 1980). According to the second version, “Including the Other in the Self,” when X empathizes with Y, X sees Y as part of what X thinks of as “me” (Wegner, 1980; Aron and Aron, 1986). According to the third version, “Seeing Aspects of the Self in the Other,” when X empathizes with Y, X sees aspects of oneself in Y (Davis et al., 1996; Cialdini et al., 1997). According to the fourth version, “Self and Other as Interchangeable Exemplars of Shared Group Identity,” when X empathizes with Y, X sees oneself and Y as interchangeable members of the same group – as equivalent exemplars of a shared group identity (Turner, 1987). Note, however, that Batson’s classification of SMH can be disputed; see the footnote 6.

^5^This scale consists of seven sets of two circles each. In the first pair, the circles are completely separated; they gradually overlap in the following pairs, and they completely overlap in the last pair. For details, see Aron et al. (1992).

^6^The group identification interpretation belongs to the fourth category (“Self and Other as Interchangeable Exemplars of Shared Group Identity”) in Batson’s classification of SMH, which we mentioned in the footnote 4. However, this does not necessarily imply that our proposal is radically different from Cialdini’s proposal that is classified in the third category (“Seeing Aspects of the Self in the Other”) by Batson. Despite Batson’s distinction between the third category and the fourth category, there are some important similarities between our proposal and Cialdini’s proposal. As we will see in “Discussion: Is Empathy-Induced Helping Egoistic or Altruistic?” for example, both blur the traditional dichotomy between egoism and altruism.

^7^It is not our claim, however, that the minimal group studies by Tajfel and colleagues and the empathy-altruism studies by Batson and colleagues are exactly the same. An important difference between them is that empathy plays a crucial role in the latter but not in the former. Our claim here is rather that there is a crucial common factor in the helping behavior in the two sets of studies; that is, group identification. For a related issue, see our distinction between “empathy-induced, group identification-driven helping behavior” and “group identification-driven helping behavior” in “The Traditional Dichotomy”.

^8^The causal interpretation is coherent with the idea by Cialdini and colleagues that the primary role of empathy is to serve as an affective signal of self-other merging: “When one feels empathic concern, it is normally due to the perspective taking that attends relationship closeness and that leads to self-other overlap. Upon experiencing empathic concern for another, then, an individual is consequently informed of a likely degree of oneness with that other, and prosocial action is more probable as a result” (Cialdini et al., 1997, p. 491).

^9^Affective mirroring might not be the same as empathy itself. At least they are different according to Batson’s (2018) classification. The latter requires the congruence of valence (e.g., both X and Y feel something negative), while the former requires the congruence of feeling (e.g., X and Y share the same feeling or similar feelings). Still, Batson does not deny that affective mirroring and empathy tend to co-occur in real-life cases.

^10^Alternatively, Reynie’s stance toward Elaine is second-personal (Darwall, 2009; Tomasello, 2016); for Reynie, Elaine’s welfare is something second-personal (*your* welfare) rather than third-personal (*her* welfare). Either way, what is crucial here is that Reynie’s EGHB toward Elaine is motivated by Reynie’s concern for something other than his first-person singular (my) welfare at the individual level.

^11^For the same reason, Alba’s GHB toward Simon is egoistic at the group level in the sense that it is motivated by Alba’s concern for Simon’s welfare in so far as Simon’s welfare is constitutive of Alba’s first-person plural welfare at the group level. One might think, however, that Alba’s concern is both egoistic and altruistic at the group level given the assumption that Alba’s community is identical with Simon’s community. Alba (egoistically) cares about her own community; in doing so she (altruistically) cares about Simon’s community at the same time. At least in our taxonomy, however, Alba’s GHB is egoistic and not altruistic at the group level. When Alba group-identifies with Simon, what she cares about is her community = Simon’s community which, for Alba, is something first-personal rather than third-personal at the group level; *our* community rather than *their* community.
